# Application of Preparative High-Speed Counter-Current Chromatography for the Separation of Two Alkaloids from the Roots of *Tabernaemontana catharinensis *(Apocynaceae)

**DOI:** 10.3390/molecules16097480

**Published:** 2011-09-02

**Authors:** Milena S. Gonçalves, Ivo J. Curcino Vieira, Rodrigo R. Oliveira, Raimundo Braz-Filho

**Affiliations:** Laboratório de Ciências Químicas, Universidade Estadual do Norte Fluminense Darcy Ribeiro, 28013-602, Campos dos Goytacazes, Rio de Janeiro, Brazil

**Keywords:** counter-current chromatography, *Tabernaemontana catharinensis*, alkaloids

## Abstract

The methanolic extract of *Tabernaemontana catharinensis* (Apocynaceae) roots, which contains alkaloids with several biological activities, was separated on a preparative scale using high-speed counter-current chromatography. The optimum solvent system was found to be a mixture of CHCl_3_-MeOH-H_2_O [5:10:6 (*v/v/v*)] and led to a successful separation of two monoterpenic indole alkaloids, voachalotine (**1**) and 12-methoxy-*N*_b_-methylvoachalotine (**2**) in approximately 4.0 hours. The alkaloids were all isolated at purities over 95%, and their structures were established on the basis of spectroscopic methods, including 1D and 2D NMR and EI/MS.

## 1. Introduction

The genus *Tabernaemontana* includes about 110 species widespread in the pantropical regions, and these species are rich in alkaloids [1,2,3,4], mostly of the monoterpenic indole type, revealing a considerable variety of carbon skeletons and novel biological activities [5].

*Tabernaemontana catharinensis* A. DC. (syn. *Peschiera catharinensis* A. DC. Miers) (Apocynaceae), popularly known as ‘milkweed’, occurs in Argentina, Paraguay, Bolivia and southern Brazil. The species has also been named *T. affinis, T. australis and T. hilariana *[1]. The alkaloids and alkaloid-containing extracts from the species show diverse biological activities: antitumoral, anti-inflammatory, analgesic [6], anticrotalic and antitumoral [7], antioxidant and antimycobacterial [8], trypanocidal [9] and antileishmanial activities [10,11].

Preliminary phytochemical investigation using classical chromatographic methods (adsorption column chromatography) led to the detection of alkaloids with acetylcholinesterase inhibitory properties in the polar extracts of the roots of *T. catharinensis* [12]. The separation of compounds present in a crude extract is often performed by repeated adsorption column chromatography. In our case, we investigate mainly polar extracts containing alkaloids compounds, which can be degraded because of the acidic nature of the silica gel. To overcome this problem, the use of high-speed counter-current chromatography (HSCCC) that does not employ a solid-phase support and thus there is no irreversible adsorption associated with the solid supports, can be an alternative to the direct application of crude extracts and provide excellent recovery of the compounds [13]. This method has been successfully applied to the analysis and separation of various natural products [14,15]. One of the advantages of HSCCC in relation to others liquid–liquid chromatography techniques are the possibility of shorter separation times. The main purpose of this contribution was to study the purification on a preparative scale of the two main monoterpenic indole alkaloids, voachalotine (**1**) and 12-methoxy-*N*_b_-methylvoachalotine (**2**), from *T. catharinensis* by HSCCC.

## 2. Experimental

### 2.1. Reagents

All organic solvents used for HSCCC were of analytical grade and purchased from Synth (São Paulo, Brazil). Water was purified by Milli-Q system (Millipore, Bedford, MA, USA). voachalotine (**1**) and 12-methoxy-*N*_b_-methylvoachalotine (**2**) standards were obtained in our Laboratory of Chemical Sciences (UENF, Brazil) by column chromatography. The identity of the isolated alkaloids were confirmed by their spectroscopic properties (^1^H- and ^13^C-NMR). NMR spectra were obtained on a Brüker Advance II 9.4 T instrument with CDCl_3_ (0.1% TMS) as solvent. UV spectra were recorded on a Shimadzu model 1800 Shimadzu software UV - PROBE and IR data on a model IR AFFINITY, SHIMADZU and GC/MS analysis were carried out on a Shimadzu CG/EM-QP-5050 SHIMADZU, at 70 eV (column DB5, 30 m).

### 2.2. Preparation of Crude Sample and Sample Solution

Roots (2.8 Kg) of *T. catharinensis* were collected at Araguari, Minas Gerais State, Brazil and authenticated by Luiza S. Kinoshita. A voucher specimen (No. UEC117862) was deposited at the Herbarium of the Unicamp University, Campinas, São Paulo State, Brazil. The air-dried and powdered roots (1.8 Kg) were exhaustively extracted (three times) with methanol (3L) at room temperature (3 days). Solvents were evaporated under reduced pressure affording the crude MeOH extract (63.2 g). The MeOH extract was submitted to silica gel column chromatography affording 16 fractions (1-16). An aliquot of fraction 12 (0.18 g) containing of mixture of alkaloids **1** and **2** was dissolved in 5 mL of a mixture consisting of 2.5 mL lower phase + 2.5 mL upper phase of the solvent system CHCl_3_-MeOH-H_2_O (5:10:6 (*v/v/v*)). 

### 2.3. Apparatus

HSCCC was carried out using a Dynamic Extractions Ltd. Model Spectrum-1000 mini high-speed counter-current chromatography unit (Berkshire, UK), containing a self-balancing three-coil centrifuge rotor equipped two preparative multiplayer coils with a total capacity of 142 mL, the internal diameter of PTFE tubing was 1.6 mm. The β-value range varied from 0.52 at internal to 0.86 at the external terminal. The speed was adjusted with a controller to an optimum speed of 1,200 rpm. The flow rate was controlled with a Knauer Smartline-1000 constant flow pump with a capacity of 5 mL/min. The sample was injected via a P.C. injection module with a 5 mL sample injection loop. The coiled column was entirely filled with the stationary phase (upper phase). Then the apparatus was rotated forward at 1,200 rpm, while the mobile phase (lower phase) was pumped into the column in a head to tail direction at a flow rate of 1.5 mL/min. After the mobile phase front emerged, 5 mL of the sample solution containing the sample of fraction 12 of the methanolic extract was injected at a flow rate 1.5 mL/min. We collected 220 fractions of 4 mL each with a Büchi fraction collector C-660, in approximately 4 h, while monitoring with a UV-Knauer detector at 300 nm.

### 2.4. Preparation of Two-Phases Solvent System

The solvent system composed of CHCl_3_-MeOH-H_2_O (5:10:6 (*v/v/v*)) was throughly equilibrated in a separatory funnel at room temperature and two phases separated shortly before use.

### 2.5. Analyses of the Alkaloids by TLC

An aliquot of fraction 12 and the collected 214 fractions were analyzed using silica gel on aluminium TLC sheets (20 cm × 20 cm, Merck) developed with a solvent mixture composed of CH_2_Cl_2_-MeOH [9:1 (*v/v*)]. The spots on the TLC sheets were observed under a UV lamp (254 nm) and revealed with sulfuric vanilin and Dragendorff reagents. Fractions of similar retention factors (*R*_F_) were combined and subimetted from the analyses NMR.

### 2.6. Structural Identification of the Alkaloids

The spectral data of the two alkaloids are as given below:

*Voachalotine* (**1**): ^1^H-NMR (400 MHz, δ_H_ in ppm): δ_H_ 7.50 (1H, br d, *J *= 7.6 Hz, H-9), 7.21 (1H, br d, *J = *8.3 and 1.1 Hz, H-11), 7.11 (1H, br d, *J = *7.9 and 1.4 Hz, H-10), 7.30 (1H, br d, *J = *8.3 Hz, H-12), 1.63 (3H, d, *J = *7.9 Hz, 3H-18), 5.34 (1H, tq, *J = *6.6 and 2.0 Hz, H-19), 4.22 (1H, dd, *J = *9.9 and 4.1 Hz, H-3), 4.33 (1H, d, *J = *6.0 Hz, H-5), 3.25 (1H, br t, *J = *3.0 Hz, H-15), 3.15 (1H, dd, *J = *15.6 and 6.3 Hz, H-6a), 2.99 (1H, br d, *J *= 15.6 Hz, H-6b), 2.02 (1H, ddd, *J = *12.6, 9.7 and 2.6 Hz, H-14a), 1.83 (1H, td, *J = *12.6 and 3.6 Hz, H-14b), 3.71 (1H, br d, *J = *11.7 Hz, H-17a), 3.62 (1H, d, *J = *11.7 Hz, H-17b), 3.60 (2H, m, H-21a and H-21b), 3.64 (3H, s, MeN-1), 3.76 (3H, s, MeO-22). ^13^C-NMR (100 MHz, δ_C_ in ppm): δ_C_ 137.4 (C-2), 105.0 (C-7), 126.0 (C-8), 138.0 (C-13), 53.4 (C-16), 137.5 (C-20), 176.4 (C-22), 48.0 (CH-3), 53.8 (CH-5), 118.3 (CH-9), 119.0 (CH-10), 121.2 (CH-11), 108.9 (CH-12), 29.9 (CH-15), 116.5 (CH-19), 22.3 (CH_2_-6), 28.4 (CH_2_-14), 63.1 (CH_2_-17), 54.0 (CH_2_-21), 12.9 (CH_3_-18), 30.3 (CH_3_N-1), 52.3 (CH_3_O-22).

*12-Methoxy-N_b_-methylvoachalotine* (**2**): ^1^H-NMR (400 MHz, δ_H_ in ppm): δ_H_ 6.28 (1H, br d, *J = *11.1 Hz, H-3), 4.96 (1H, d, *J = *6.4 Hz, H-5), 7.27 (1H, d, *J = *7.0 Hz, H-9), 7.25 (1H, t, *J = *7.0 Hz, H-10), 6.93 (1H, d, *J = *7.0 Hz, H-11), 3.31 (1H, m, H-15), 5.60 (1H, q, *J = *6.4 Hz, H-19), 3.96 (1H, m, H-6a), 3.30 (1H, m, H-6b), 2.40 (1H, m, H-14a), 1.86 (1H, m, H-14b), 3.78 (1H, m, H-17a), 3.13 (1H, m, H-17b), 5.31 (1H, br d, *J = *15.8 Hz, H-21a), 3.90 (1H, m, H-21b), 1.82 (3H, d, *J = *6.4 Hz, H-18), 4.18 (3H, s, MeN-1), 3.35 (3H, s, MeN-4), 4.18 (3H, s, MeO-12), 3.99 (3H, s, MeO-22). ^13^C-NMR (100 MHz, δ_C_ in ppm): δ_C_ 132.4 (C-2), 101.4 (C-7), 126.9 (C-8), 147.6 (C-12), 126.9 (C-13), 55.0 (C-16), 127.6 (C-20), 172.8 (C-22), 56.5 (CH-3), 64.2 (CH-5), 111.7 (CH-9), 120.9 (CH-10), 103.1 (CH-11), 29.4 (CH-15), 119.9 (CH-19), 19.0 (CH_2_-6), 27.7 (CH_2_-14), 62.2 (CH_2_-17), 64.0 (CH_2_-21), 12.1 (CH_3_-18), 32.9 (CH_3_N-1), 48.7 (CH_3_N-4), 55.3 (CH_3_O-12), 52.8 (CH_3_O-22).

## 3. Results and Discussion

The selection of the two-phase solvent system is the most important and critical step in performing HSCCC. One rule for HSCCC mentioned in the literature [16] was the need to find systems with *K`* (partition coefficient) values of the target compounds in a proper range: the suitable *K` *values for HSCCC are 0.5 ≤ *K *≤ 1.0. In the present study, the suitable solvent system for HSCCC separation was developed according this rule considering the properties of the target compounds (monoterpenic indole alkaloids). Thus CHCl_3_-MeOH-H_2_O with different volume ratios were tested for HSCCC separation of these compounds.

**Figure 1:**
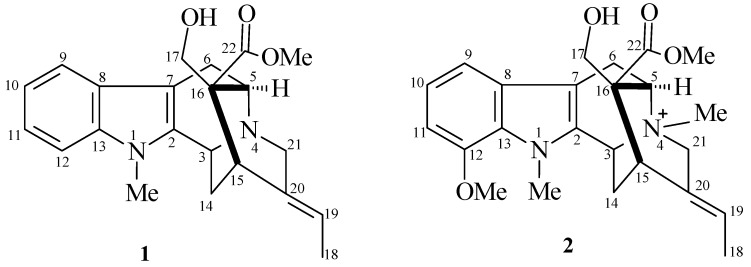
Structure of the isolated alkaloids: voachalotine (**1**) and 12-methoxy-*N*_b_-methylvoachalotine (**2**).

**Figure 2:**
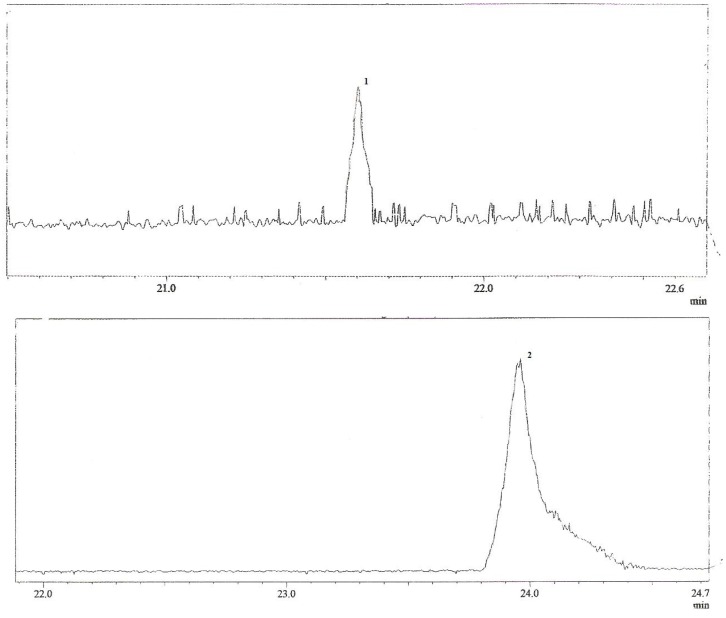
CG of alkaloids voachalotine (**1**) and 12-methoxy-*N*_b_-methylvoachalotine (**2**).

A series of experiments was performed to determine a suitable two-phase solvent system for HSCCC [17]. Small amounts of the MeOH fraction (5 mg) were dissolved in 5 μL of two immiscible liquid phases consisting of organic solvents and water. From this mixture, 1.5 μL of the upper phase and 1.5 μL of the lower phase were spotted onto TLC plates and eluted with a solvent system containing organic solvents and water [17]. After elution, plates were dried and observed under UV light (254 nm) and revealed with sulfuric vanillin and Dragendorff reagents in order to compare the intensity of the spots containing alkaloids in each phase. TLC analyses allowed estimation of the distribution of the alkaloids between the two phases. A 5:10:6 (*v/v/v*) mixture of CHCl_3_-MeOH-H_2_O gave the best results, with the alkaloids of interest being almost equally distributed between the two phases (partition coefficient value *K*` of approximately 1). A good chromatographic resolution was obtained with the lower phase of this solvent system, with *R*_F_ values of 0.7 and 0.6 for compounds **1**–**2**, respectively. The relatively high proportion of MeOH in the solvent mixture and the *R*_F_ values of the compounds indicate the medium or high polarity of the *T. catharinensis* alkaloids*. *Thus the lower phase was chosen as mobile phase and the upper phase was used as stationary phase for the CCC separation of the MeOH fraction of the methanolic extract of *T. catharinensis*. Under the conditions used, the retention of the stationary phase in the HSCCC was 82%. After the HSCCC separation, the collected fractions were monitored by UV-detector (300 nm) and combined based on TLC. Spectroscopic analyses allowed us to identify two main compounds, identified by comparison with literature data [18,19] as voachalotine (**1**) and 12-methoxy-*N*_b_-methylvoachalotine (**2**) (Figure 1) [20,21]. The identity and purity of the isolated compounds were also checked by TLC analyses using authentic samples from a collection of our laboratory. The alkaloid voachalotine (**1**) was isolated with purity approximately 95%, whereas 12-methoxy-*N*_b_-voachalotine (**2**) was obtained with purity approximately 97%. The purity of the alkaloids **1** and **2** was also confirmed by CG/MS (Figure 2).

**Figure 3:**
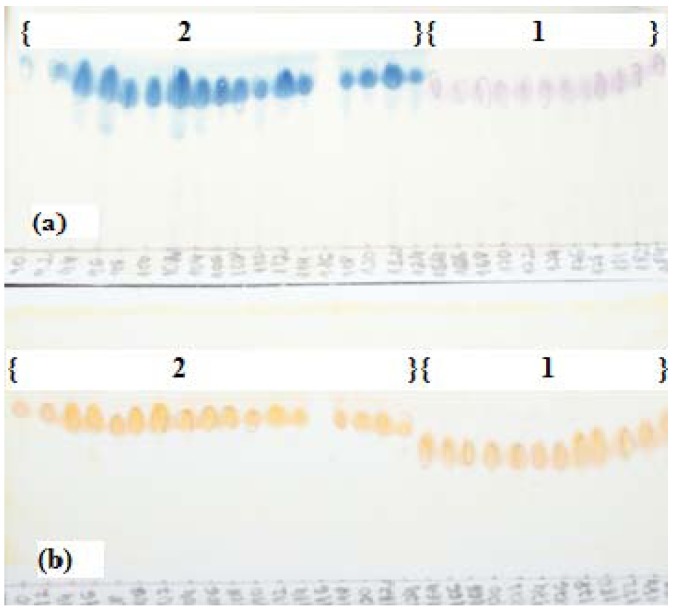
TLC monitoring of fractions from the HSCCC separation of fraction 12 from silica gel chromatography of the MeOH extract of *T. catharinensis *roots: voachalotine (**1**) (fractions 90-124) and 12-methoxy-*N*_b_-methylvoachalotine (**2**) (fractions 164-186). TLC silica gel plates were eluted in CH_2_Cl_2_-MeOH (9:1 (*v/v*)) and the chemical detection was done by spraying sulfuric vanillin (**a**) and Dragendorff reagents (**b**).

Figure 3 shows the TLC analysis of the purified fractions from the HSCCC separation of fraction 12 of the MeOH extract of *T. catharinensis *roots: voachalotine (**1**, fractions 90-124) and 12-methoxy-*N*_b_-methylvoachalotine (**2**, fractions 164-186). TLC silica gel plates were eluted in CH_2_Cl_2_-MeOH (9:1 (*v/v*)) and the chemical detection was done by spraying sulfuric vanillin (**a**) and Dragendorff reagents (**b**).

This approach led to the isolation of the two alkaloids in approximately 4.0 h. The alkaloid 12-methoxy-*N*_b_-methylvoachalotine (**2**) was the first compound to be eluted (after 2.4 h), because of its lower polarity compared to voachalotine (**1**). Thus, the separation of these alkaloids seems to depend on the methoxyl group attached to the benzene ring and the methyl group attached to the nitrogen atom at position 4 in alkaloid 12-methoxy-*N*_b_-methylvoachalotine (**2**). Our results showed that the conditions used provided a very efficient method for the separation of the alkaloids from *T. catharinensis *roots.

Preliminary assays with alkaloid 12-methoxy-*N*_b_-methylvoachalotine (**2**) exhibited a significative anti acetylcholinesterase effect (results not shown). Since **1**–**2** were obtained in good amounts and purity, without the need of further steps of purification, we can conclude that the HSCCC conditions used in our work fits well with our objective of obtaining these compounds to perform future acetylcholinesterase inhibitors tests.

## 4. Conclusions

The results of our studies clearly demonstrate the potential of HSCCC for the preparative isolation of voachalotine (**1**) and 12-methoxy-*N*_b_-methylvoachalotine (**2**) from the roots of *T. catharinensis*. In particular, preparative HSCCC with its rapid separation and minimum solvent consumption offers a very efficient method for the separation and purification of monoterpenic indole alkaloids.
